# Efficacy of traditional Chinese medicine prescription in treatment of COVID-19: A prospective cohort study

**DOI:** 10.1097/MD.0000000000043285

**Published:** 2025-07-18

**Authors:** Feihua Huang, Ying Zhou, Cheng Jiang, Yifan Wang, Jinguo Cheng, Xiaoyang Lin, Liping Chen, Chunxian Peng, Guangqiang Pan, Xiaodi Chen, Li Yin, Kequn Chai

**Affiliations:** a Tongde Hospital of Zhejiang Province Affiliated to Zhejiang Chinese Medical University, Hangzhou, China; b Key Laboratory of Cancer Prevention and Therapy Combining Traditional Chinese and Western Medicine of Zhejiang Province, Zhejiang Academy of Traditional Chinese Medicine, HangZhou, China; c First Affiliated Hospital of Wenzhou Medical University, Wenzhou, China; d First People’s Hospital of Wenling, Wenling, China; e Traditional Chinese Medicine Hospital of Lishui City, Lishui China; f People’s Hospital of Quzhou City, Quzhou, China; g People’s Hospital of Ruian City, Ruian, China; h Central Hospital of Yiwu City, Yiwu, China; i Fourth Affiliated Hospital of Zhejiang University School of Medicine, Yiwu, China.

**Keywords:** clinical symptom, Coronavirus disease 2019, efficacy, partial least squares discriminant analysis, traditional Chinese medicine

## Abstract

**Background:**

The purpose of this study was to estimate the efficacy of traditional Chinese medicine (TCM) prescription in the treatment of coronavirus disease 2019 (COVID-19).

**Methods:**

A multicenter prospective cohort study at 7 hospitals was conducted. The COVID-19 inpatients were divided into 2 groups. The control group received conventional treatment, and the TCM group received conventional treatment in combination with TCM prescription (Huashi Xuanfei decoction, Jiedu Xiefei decoction and Jianpi Bufei decoction). The 24 clinical symptoms of each patient were surveyed during 7 periods. The partial least squares discriminant analysis algorithm and Mann–Whitney *U* test were applied to systematical analyzing the differences in clinical symptoms between the 2 groups.

**Results:**

A total of 38 and 77 cases were included for data analysis in the control and TCM groups, respectively. There were significant differences in scores of fever between the control and TCM groups on days 2 to 4 (1.00 vs 0.29, *P = *.003) and days 6 to 8 (0.53 vs 0.07, *P = *.024). The scores of poor appetite had significant differences between the control and TCM groups on days 0 to 1 (0.08 vs 0.51, *P = *.000), days 2 to 4 (0.05 vs 0.39, *P = *.001), days 20 to 22 (0.24 vs 0.05, *P = *.004) and days 27 to 29 (0.14 vs 0.03, *P = *.044). The scores of expectoration had significant differences on days 0 to 1 (0.32 vs 0.83, *P = *.000) and days 2 to 4 (0.24 vs 0.59, *P = *.001).

**Conclusion:**

The superiority of TCM prescription in improving symptoms of fever, poor appetite, and expectoration was demonstrated. The treatment regimen of using conventional treatment in combination with TCM prescription can significantly improve the clinical symptoms of COVID-19.

## 1. Introduction

The coronavirus disease 2019 (COVID-19) had broken out in early February 2020 and quickly spread around the world. It has been confirmed that COVID-19 can be transmitted from person to person, posing a serious threat to human health.^[1]^ The most prominent symptoms of COVID-19 include fever, cough, fatigue, acute respiratory distress syndrome, and in severe cases, death.^[2,3]^ Up to now, the cumulative number of confirmed COVID-19 cases has exceeded 500 million in over 200 countries, and >6 million people have died.

Since the outbreak of COVID-19, unprecedented public health interventions have been taken in many countries to curb the spread of the virus by the principles of early detection, early reporting, early isolation, and early treatment. The treatment of COVID-19 mainly includes supportive care, respiratory assistance, antiinfective, and glucocorticoid therapy. Great efforts have focused on testing antiviral drugs, and several antiviral drugs such as alpha-interferon, ribavirin and arbidol have been recommended. However, there is still a lack of specific drugs for COVID-19 according to the World Health Organization. Furthermore, the challenges have increased dramatically on account of the rapid mutation of the coronavirus. In the past 2 decades, the epidemics of coronaviruses including Severe Acute Respiratory Syndrome coronavirus, Middle East Respiratory Syndrome coronavirus, and novel coronavirus all broken out successively with high mortality rates.^[3]^ Nevertheless, the development of new targeted drugs is typically a time-consuming and arduous project.^[4]^

Different from western medicine, traditional Chinese medicine (TCM) achieves antivirus action with multiple components and targets. It not only deals with etiological factors to eradicate pathogenic microorganisms but also helps the body restore from the disease through overall regulations, including balanced immune response, improved hematopoiesis and coagulation systems, enhanced functions of liver and heart, increased nutrient intake and lipid metabolism.^[5]^ In ancient China, TCM had been used for effective treatment of infectious diseases.^[6]^ After more than 2000 years of inheritance, rich experience in infectious disease treatment has been accumulated from abundant clinical observations. During the Severe Acute Respiratory Syndrome epidemic, a certain positive effect of TCM on the absorption of pulmonary infiltration has been demonstrated.^[7]^ Till now, substantial pieces of evidence based on extensive clinical practices have suggested that TCM plays an important role in the treatment of infectious diseases in China.

As the battle against COVID-19 progressed, the Chinese treatment regimen of using conventional treatment in combination with TCM has drawn increasing attention from the international community. Since February 2020, TCM have being applied to a considerable number of COVID-19 pneumonia cases in China.^[5,8–11]^ Till now, TCMs like Qingfei Paidu decoction, Sanwu Huangqin decoction, and Lianhua Qingwen granule have made vast contributions to the treatment and rehabilitation of COVID-19.^[12–15]^ In addition, many systematic reviews and meta-analysis studies have shown that TCM could significantly improve the clinical symptoms of COVID-19.^[16–18]^ However, doubts about TCM still exist in many countries. Since the coronaviruses already identified might only be the tip of the iceberg, with potentially more novel and severe coronaviruses events to be revealed,^[3]^ it is urgent for the global community to have a more detailed understanding of the efficacy of TCM in dealing with coronaviruses, especially COVID-19. More high-quality evidence is needed to systematically analyze the improvement of TCM on clinical symptoms of COVID-19 during both treatment stage and convalescent stage.

In this research, the efficacy of TCM prescription in improving clinical symptoms of COVID-19 was explored by a multicenter prospective cohort study. The COVID-19 inpatients were divided into 2 groups. The control group received conventional treatment, and the TCM group received conventional treatment in combination with TCM prescription. The 24 clinical symptoms of each patient were surveyed during 7 periods. The partial least squares discriminant analysis (PLS-DA) algorithm and Mann–Whitney *U* test were applied to systematical analyzing the differences in clinical symptoms between the 2 groups. This study provided straightforward and comprehensive evidence for the efficacy of TCM prescription in improving clinical symptoms of COVID-19 infection during different periods.

## 2. Methods

### 2.1. Study design

This was a multicenter prospective cohort study at 7 government designated COVID-19 hospitals in Zhejiang province of China. The inpatients receiving COVID-19 treatment from February 3, 2020 to March 19, 2020 at 7 centers were investigated. The confirmed COVID-19 cases must meet one of the following etiological evidence: real-time fluorescence reverse transcription-polymerase chain reaction detection of novel coronavirus nucleic acid positive; virus gene sequencing was highly homologous to the known novel coronavirus. Patients who met the following criteria were excluded: age below 18 years or above 85 years; women in pregnancy and lactation; patients who had severe cognitive impairment or psychiatric disorders; patients who were allergic to TCM or its ingredients. Before the trial begins, all the patients or their legal representatives voluntarily agreed to participate by giving written informed consent approved by the ethic committee.

The eligible candidates were randomly divided into 2 groups. All the patients in the 2 groups received the same therapeutic regimen according to their disease condition, including bed rest, balance of water and electrolyte to maintain the stability of the internal environment, given effective oxygen therapy measures timely, and antiviral drug therapy. The antiviral therapy was alpha-interferon (5 million U or equivalent per dose for adults, 2 mL of sterilize water for injection was adde, aerosol inhalation twice daily), lopinavir/ritonavir (200 mg/50 mg/tablet for adults, 2 tablets each time, 2 times a day) and ribavirin (recommended to be combined with interferon or lopinavir/ritonavir, 4.0 g on the first day/1.2 g on the next days or 8 mg/kg, 3× a day intravenous infusion). The control group received the above treatments, and the TCM group received additional TCM prescription in combination of these treatments. Except for the TCM prescription, all the other therapeutic regimen in the 2 groups were consistent. In the TCM group, the TCM prescriptions were applied following the theory of pattern differentiation. The cold-dampness stagnating in the lung pattern and epidemic toxins blocking the lung pattern were incorporated into this study. During the clinical treatment stage, 2 TCM prescriptions named Huashi Xuanfei decoction and Jiedu Xiefei decoction were applied to the 2 patterns, respectively. As the treatment progressed, the third TCM prescription named Jianpi Bufei decoction was administrated when the patient transformed into lung and spleen qi deficiency pattern. The administration of 3 TCM prescriptions was one package daily, 150 mL after decocting, divided into 2×, equally in the morning and evening, taken before the meal.

### 2.2. Data collection

The baseline information of each patient, including gender, age, heart rate, blood pressure, and respiratory rate, was recorded. In addition, the severity of disease at the time of initial enrollment was also diagnosed patient by patient, which was classified into 4 grades, including light, ordinary, heavy duty, and critical types. Under the guidance of TCM theory, a TCM symptom form was then designed for evaluating the clinical symptoms of COVID-19. The TCM symptom form contained 24 TCM symptoms, including 4 primary symptoms, 7 secondary symptoms, and 13 concurrent symptoms. The names, descriptions, and scoring standards of 24 TCM symptoms are outlined in Table 1. Throughout the study, the 24 TCM symptoms were scored after a comprehensive diagnosis of each patient during 7 periods, including P1 in the screening stage, P2 to P5 in the treatment stage, and P6 to P7 in the convalescent stage. The categories and descriptions of the 7 periods are outlined in Table 2. Totally, the scores of 24 TCM symptoms (V1–V24) in 7 periods (P1–P7) were acquired. All the severity assessment, pattern differentiation, treatment, and TCM symptoms scoring were conducted by senior TCM physicians in corresponding hospitals after unified training. The details of standards for severity assessment and treatment can be found in “Diagnostic and treatment protocols of novel coronavirus pneumonia (Trial version 5)” issued by Chinese National Health Commission. The pattern differentiation and scoring standards of each symptom were developed based on the “Guidelines for clinical research of new TCM (version 2002).”

**Table 1:** Names, descriptions, and scoring standards of 24 traditional Chinese medicine symptoms.

Number	Name	Description	Score
Primary symptom			
V1	Fever	No fever	0
		37.5–38.0°C	2
		38.1–39°C	4
		Above 39°C	6
V2	Cough	No cough	0
		Intermittent or occasional cough, which does not affect daily life	2
		Cough during the day or at night, but still can persist normal life	4
		Cough frequently day and night, which affects sleep and rest	6
V3	Fatigue	No fatigue	0
		Fatigue after activity	2
		Fatigue after slight activity	4
		Fatigue when sleep and rest	6
V4	Chest tightness and short of breath	No chest tightness and short of breath	0
		Chest tightness and short of breath after activity	2
		Chest tightness and short of breath after slight activity	4
		Chest tightness and short of breath when sleep and rest	6
Secondary symptom			
V5	Unconsciousness	Clear consciousness	0
		Unconsciousness	1
		Disturbance of consciousness	2
		Lethargy and coma	3
V6	Diarrhea and loose stool	No diarrhea or loose stool	0
		Unshaped stool, 1–2 times/d	1
		Muddy stool, 3–4 times/d	2
		Watery diarrhea stool, 5 times/d or more	3
V7	Throat pain	No throat pain	0
		Pharyngeal discomfort, slight pain	1
		Throat pain is obvious, but still can persist normal diet	2
		Throat pain is obvious, which affects diet	3
V8	Expectoration	No expectoration	0
		Expectoration 10–50 mL/d	1
		Expectoration 50–100 mL/d	2
		Expectoration more than 100 mL/d	3
V9	Muscular soreness	No muscular soreness	0
		Sligh muscular soreness	1
		Obvious muscle soreness, but still can persist flexion and extension	2
		Obvious muscle soreness, which affects flexion and extension	3
V10	Nasal obstruction	No nasal obstruction	0
		Sligh nasal obstruction	1
		Obvious nasal obstruction, but still can breathe through nose	2
		Obvious nasal obstruction, must breathe through mouth	3
V11	Runny nose	No runny nose	0
		Occasional runny nose	1
		Sometimes runny nose	2
		Persistent runny nose	3
Concurrent symptom			
V12	Chest pain	No	0
		Yes	1
V13	Headache	No	0
		Yes	1
V14	Cold limbs	No	0
		Yes	1
V15	Dizziness or vertigo	No	0
		Yes	1
V16	Nausea and vomiting	No	0
		Yes	1
V17	Poor appetite	No	0
		Yes	1
V18	Stomach bilge or abdominal distention	No	0
		Yes	1
V19	Dry mouth	No	0
		Yes	1
V20	Spontaneous perspiration	No	0
		Yes	1
V21	Anxiety	No	0
		Yes	1
V22	Insomnia and dreaminess	No	0
		Yes	1
V23	Throat wheezing	No	0
		Yes	1
V24	Blood in sputum	No	0
		Yes	1

**Table 2:** Categories and descriptions of 7 periods.

Category	Number	Description
Screening stage	P1	Days 0–1
Treatment stage	P2	Days 2–4
	P3	Days 6–8
	P4	Days 9–11
	P5	Days 13–15
Convalescent stage	P6	Days 20–22
	P7	Days 27–29

Day 0 represented the date when the patient having positive result of novel coronavirus nucleic acid test.

### 2.3. Statistical methods

First, the baseline information between the 2 groups was compared using the χ^2^ test (gender, severity of disease and TCM pattern differentiation) and the *t* test (age, heart rate, blood pressure, and respiratory rate). Then, the 24 variables of each patient during 7 periods were expanded in the patient direction to build matrix X (I × 168, I represents the number of patients, 168 = 24 × 7). The grouping information was encoded as matrix Y (I × 1) by assigning the control group to 0 and the TCM group to 1. The PLS-DA model was established to analyze the difference between the 2 groups and the number of principal components was selected using 7-fold cross-validation. The effectiveness of the PLS-DA model was estimated using R2Y and Q2. The influential variables between the 2 groups were screened using regression coefficients and Variable Importance in Projection (VIP) values. Mann–Whitney *U* test was applied to verifying the results of PLS-DA. PLS-DA was performed using SIMCA-P + (Umetrics, Sweden, version 14.1). χ^2^ test, *t* test, and Mann–Whitney *U* test were performed using MINITAB (Minitab Inc., version 14.1).

## 3. Results

### 3.1. Patient inclusion

**Figure 1.**
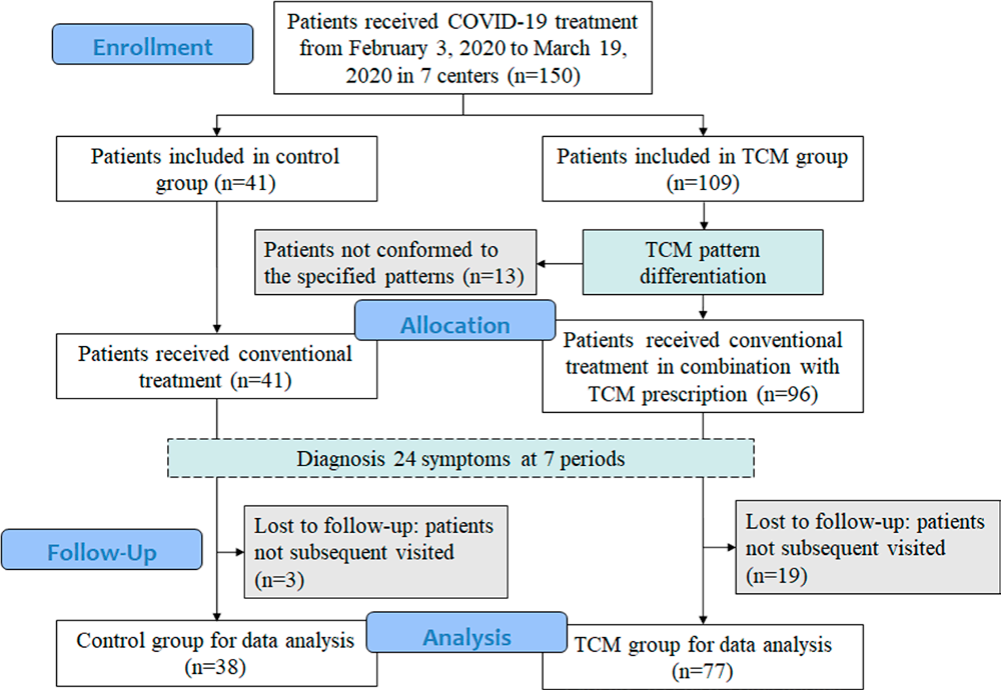
Flowchart of case selection.

From February 3, 2020 to March 19, 2020, a total of 150 inpatients receiving COVID-19 treatment were identified in 7 centers, among which 41 patients were included in control group and 109 were included in TCM group. No case was excluded because of age, pregnancy, lactation, severe cognitive impairment, psychiatric disorders, or allergy. In the control group, 31 patients conformed to the cold-dampness stagnating in the lung pattern and 10 patients conformed to the epidemic toxins blocking the lung pattern. All the 41 patients conforming to the 2 specified patterns were included. While in the TCM group, 67 patients conformed to the cold-dampness stagnating in the lung pattern, 29 patients conformed to the epidemic toxins blocking the lung pattern, and 13 patients conformed to the lung and spleen qi deficiency pattern. 96 patients conforming to the 2 specified patterns were included, while 13 patients conforming to other patterns were excluded. The scores of 24 TCM symptoms in 7 periods were recorded after comprehensive diagnosis of each patient. As the treatment progressed, most inpatients were discharged from the hospital. The scores of 24 TCM symptoms were followed up when they were subsequently visited. 3 cases in the control group and 19 cases in the TCM group were excluded since the patients had not subsequently visited. Finally, 38 cases in the control group and 77 cases in the TCM group were included for data analysis. The flowchart of case selection is shown in Figure 1. The gender, age, heart rate, blood pressure, respiratory rate, severity of disease, and TCM pattern differentiation between the control and TCM groups have no statistical differences at baseline (*P* > .05). The details of baseline information can be found in our previous work.^[19]^

### 3.2. Overview of symptoms profile

**Figure 2.**
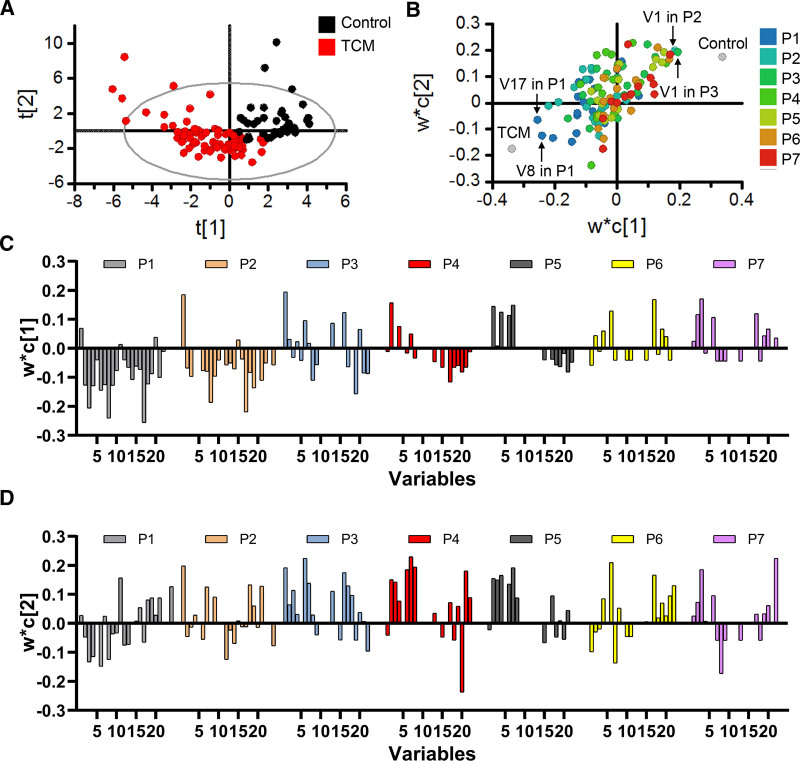
Overview of symptoms profile based on the PLS-DA model. (A) Scores plot. (B) Loadings scatter plot. (C) w*c [1] loadings plot. (D) w*c [2] loadings plot. PLS-DA = partial least squares discriminant analysis.

To obtain an overview of the symptoms profile between the 2 groups, the PLS-DA model was established based on X (115 × 168) and Y (115 × 1). To make individual variables more comparable, the data set was scaled by unit variance for unifying the influence of each variable. The optimal number of principal components was 2. The R2Y represents the cumulative fraction of Y variation modeled in the 2 components and the Q2 represents the cumulative overall cross-validated R2 for the 2 components. The R2Y and Q2 were 0.768 and 0.413, respectively. The scores plot of the PLS-DA model is shown in Figure 2A. The control group and TCM group tended to cluster in different regions of the scores plot, indicating that there were different characteristics of symptoms profile between the 2 groups.

The w*c loading weights plot is a superimposition of the w* plot and the c plot, which displays the relation between X and Y. The loadings scatter plot, w*c [1] loadings plot, and w*c [2] loadings plot of the PLS-DA model are shown in Figure 2B–2D. The V1 in P2 and V1 in P3 were situated in the vicinity of the control group with positive values in both w*c [1] and w*c [2] loadings. The result showed that the recovery of fever in the TCM group was faster than that in the control group, particularly in P2 and P3. Most variables in P1 were situated in the vicinity of the TCM group with negative values in both w*c [1] and w*c [2] loadings, especially V17 and V8. The result showed that some symptoms in the TCM group were more severe than those in the control group at baseline, but as the treatment progressed the differences decreased.

### 3.3. Screening of influential symptoms

**Figure 3.**
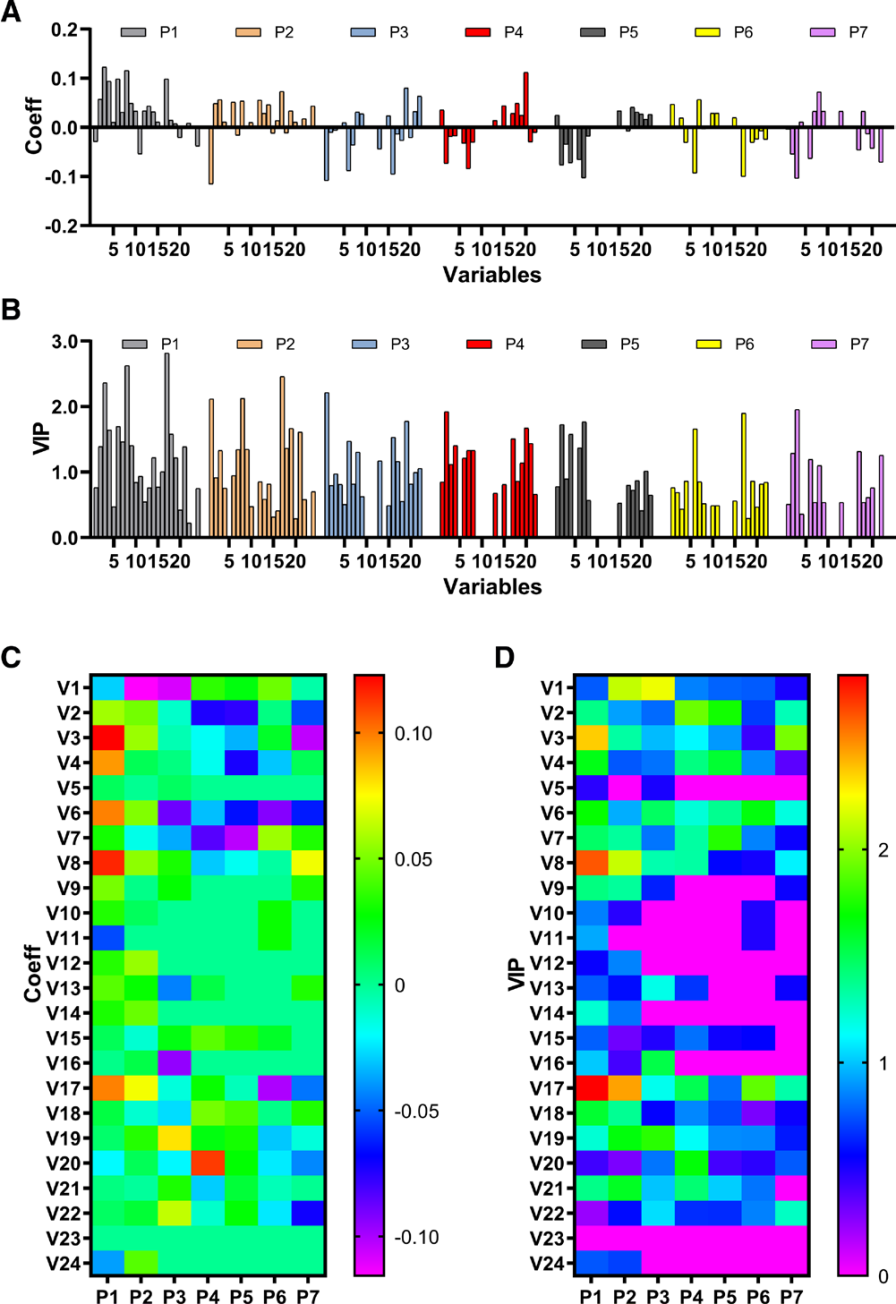
Screening of influential symptoms based on the PLS-DA model. (A) Regression coefficients plot. (B) VIP plot. (C) Heat map plot of regression coefficients. (D) Heat map plot of VIP. PLS-DA = partial least squares discriminant analysis, VIP = Variable Importance in Projection.

The influential symptoms were screened according to the regression coefficients and VIP values of the PLS-DA model. The regression coefficients express how strongly Y is correlated to the systematic part of each of the X variables. The VIP values summarize the importance of the variables both to explain X and to correlate to Y. The regression coefficients plot and VIP plot are shown in Figure 3A and 3B. To make the individual variables in different periods more comparable, the heat map plots of regression coefficients and VIP values are shown in Figure 3C and 3D. The V1 in P2, V1 in P3, V17 in P1, and V8 in P1 were identified as influential variables since they had both high absolute values of regression coefficients and high values of VIP.

The regression coefficients of V1 in P2 and V1 in P3 were −0.116 and −0.108, while the VIP values were 2.12 and 2.21, respectively. The V1 in P2 and V1 in P3 had negative values of regression coefficients as well as high VIP values, indicating the fever scores of the TCM group in P2 and P3 were observably lower than those of the control group. As the treatment progressed, the body temperature in both 2 groups returned to the normal level, thus the V1 became not influential in P4 to P7. The superiority of TCM in rapid decreasing body temperature was shown.

The regression coefficients of V17 in P1 and V8 in P1 were 0.0986 and 0.116, while the VIP values were 2.82 and 2.63, respectively. The V17 in P1 and V8 in P1 had positive values of regression coefficients as well as high VIP values, indicating the symptoms poor appetite and expectoration of the TCM group were observably grievous than those of the control group before the treatment. The decreased regression coefficients and VIP values of V17 and V8 implied that the differences between the 2 groups tended to be slight. In addition, the regression coefficients of V3, V4, and V6 also observed the same tendency. The advantage of TCM in promoting recovery of the symptoms, especially poor appetite and expectoration, was demonstrated.

### 3.4. Verification of influential symptoms

**Figure 4.**
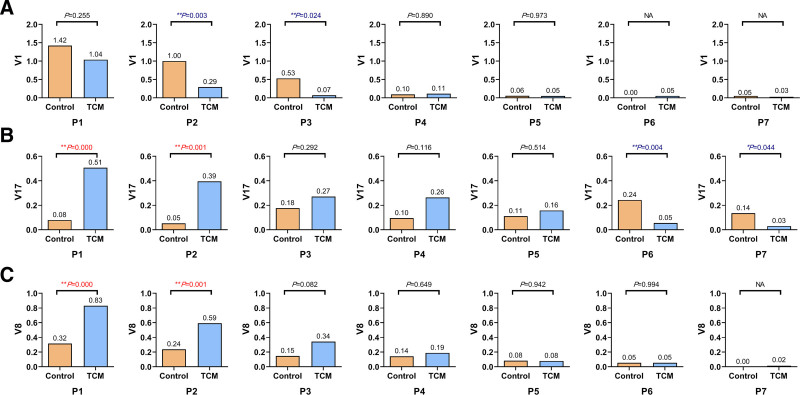
Bar plots of average score and Mann–Whitney *U* test results of influential symptoms. (A) Fever. (B) Poor appetite. (C) Expectoration. *P* values in red represented the symptom score in TCM group higher than those in control group; *P* values in blue represented the symptom score in TCM group lower than those in control group. *P < .05; **P < .01; NA: *P* value was not available because of too much zeros. TCM = traditional Chinese medicine.

In order to verify the results of PLS-DA, the differences in influential symptoms between the 2 groups were assessed using Mann–Whitney *U* test. The V1 represented the score of fever, a primary symptom, which was scored as 0, 2, 4, or 6. The bar plots of the average score and the Mann–Whitney *U* test results of V1 in 7 periods are shown in Figure 4A. As revealed by Figure 4A, the difference in fever scores between the 2 groups was not significant in P1 (*P* > .05). In P2 and P3, the scores in the TCM group decreased quickly, but the scores in the control group decreased slowly. Therefore, significant statistical differences in fever scores between the control and TCM groups were observed in P2 and P3 (P2: 1.00 vs 0.29, *P = *.003; P3: 0.53 vs 0.07, *P = *.024). As the treatment progressed, the fever scores in the control group were also prone to 0, hence the difference between the 2 groups tended to be not significant again in P4 to P5 (*P* > .05). In P6 and P7, most patients in both 2 groups recovered from fever and scored as 0, consequently, the *P* value of the Mann–Whitney *U* test was not available. The proportions of fever patients (scored not as 0) in P1 to P7 were 47%, 37%, 21%, 9.5%, 2.8%, 0.0%, and 0.0% in the control group, and 35%, 7.9%, 1.4%, 3.8%, 1.3%, 1.4%, and 1.5% in the TCM group. In the TCM group, the proportion of fever patients quickly decreased from 35% to 7.9% in P2, and to 1.4% in P3. But in the control group, the proportion of fever patients sustained above 10% until P4 (37% in P2, and 21% in P3). The results of univariate data analysis were consistently in agreement with the results of PLS-DA. The advantage of TCM for rapid promoting the recovery of fever was demonstrated.

The V17 represented the score of poor appetite, a concurrent symptom, which was scored as 0 or 1. The bar plots of the average score and the Mann–Whitney *U* test results of V17 in 7 periods are shown in Figure 4B. Being different from V1, the V17 had significant statistical differences between control and TCM groups in P1 and P2 (P1: 0.08 vs 0.51, *P = *.000; P2: 0.05 vs 0.39, *P = *.001), indicating the symptom poor appetite of TCM group was more severe than that of the control group at baseline. As the treatment progressed, the symptom improved gradually in the TCM group but had no observable improvement in the control group. Therefore, the difference between the 2 groups tended to be not significant in P3 to P5 (*P* > .05). In P6 and P7, the symptom kept improving in the TCM group, hence the difference between the control and TCM groups became significant again (P6: 0.24 vs 0.05, *P = *.004; P7: 0.14 vs 0.03, *P = *.044). The advantage of TCM for promoting the recovery of appetite was shown.

The V8 represented the score of expectoration, a secondary symptom, which was scored as 0, 1, 2, or 3. The bar plots of the average score and the Mann–Whitney *U* test results of V8 in 7 periods are shown in Figure 4C. Being the same as V17, the V8 had significant statistical differences between the control and TCM groups in P1 and P2 (P1: 0.32 vs 0.83, *P = *.000; P2: 0.24 vs 0.59, *P = *.001), indicating the symptom expectoration of the TCM group was more severe than that of the control group at baseline. As the treatment progressed, the symptom improved in both 2 groups, but the improvement was faster in the TCM group. The difference became not significant in P3 to P6 (*P* > .05). In P7, the symptom expectoration of most patients in both 2 groups was recovered and scored as 0, consequently, the *P* value of the Mann–Whitney *U* test was not available. The advantage of TCM for improving symptom expectoration was shown.

## 4. Discussion

COVID-19 belongs to the plague in TCM with the etiology of epidemic factor exposure. Based on TCM theory, the patients infected with COVID-19 can be classified to 4 patterns, including cold-dampness stagnating in the lung pattern, epidemic toxins blocking the lung pattern, internal closure and external decomposition pattern, and lung and spleen qi deficiency pattern. Among these, the third pattern is usually a critical type. The clinical manifestations of this pattern were dyspnea, frequent asthma or mechanical ventilation, dysphoria, cold limbs due to sweat, accompanied by dizziness, thick and greasy coating or dryness, purple and dark tongue, floating pulse without roots, et al Based on the results of pattern differentiation, no patient was classified to the third pattern both at the time of initial enrollment and during the treatment process. The fourth pattern was usually occurred during the recovery stage. The main clinical manifestations were shortness of breath, lassitude and fatigue, poor appetite with nausea and vomiting, and abdominal fullness, et al Because of the different states of the fourth pattern compared with other patterns, it was not included into this study at the time of initial enrollment. Finally, the first 2 patterns at the time of initial enrollment were included.

The TCM prescriptions were applied following the theory of pattern differentiation. As the treatment progressed, the patterns might transform into each other. At the end of the treatment, all the patients transformed into the lung and spleen qi deficiency pattern. Huashi Xuanfei decoction, Jiedu Xiefei decoction and Jianpi Bufei decoction were applied to cold-dampness stagnating in the lung pattern, epidemic toxins blocking the lung pattern and lung and spleen qi deficiency pattern, respectively. All the TCM prescriptions were formulated by a TCM expert group based on the local drug use characteristics in Zhejiang province and the classical prescriptions in ancient books, including “Prescriptions People’s Welfare Pharmacy,” “Item Differentiation of Warm Febrile Diseases,” and “Origin of Medicine.” In this study, the pattern differentiation of every patient was conducted and corresponding TCM prescription was administrated in 7 periods. For example, Jiedu Xiefei decoction was applied to a patient who was diagnosed as epidemic toxins blocking the lung pattern at the time of initial enrollment. On the next period, this patient might transform into the cold-dampness stagnating in the lung pattern, consequently Huashi Xuanfei decoction was applied. If the patient transformed into the lung and spleen qi deficiency pattern, the third prescription named Jianpi Bufei decoction was administrated. In consideration of the composite treatment of TCM, the patients all used at least 2 kinds of TCM prescriptions. Therefore, the effect of individual TCM prescription was not investigated.

The main clinical manifestations of cold-dampness stagnating in the lung pattern were fever, fatigue, poor appetite, cough, expectoration, and so on. Accordingly, the Huashi Xuanfei decoction was formulated with the main objectives of clearing heat and dampness, dispersing lung and phlegm, dispersing evil and detoxifying. The primary basis of the Huashi Xuanfei decoction was Huoxiang Zhengqi powder in “Prescriptions People’s Welfare Pharmacy” and Yingqiao powder in “Item Differentiation of Warm Febrile Diseases.” Huashi Xuanfei decoction consisted of several TCMs including Radix Tetrastigmae (San Ye Qing), Fructus Forsythiae (Lian Qiao), Lonicerae Japonicae (Jin Yin Hua), Herba Pogostemonis (Huo Xiang), and so on. Radix Tetrastigmae was a sovereign drug, which had been proved to have plentiful bioactivities such as antivirus, antiinflammatory, antioxidant, antibacterial, and immunoregulatory.^[20]^ Fructus Forsythiae and Lonicerae Japonicae have an obvious effect on clearing heat and detoxification. In addition, the special fragrance in Herba Pogostemonis can effectively enhance appetite and has been widely employed to treat stress and fatigue. A network pharmacology study showed that Huashi Xuanfei decoction may improve the symptoms of COVID-19 through multitargets and multipathways. The herbs-targets network of Huashi Xuanfei decoction mainly contained 70 corresponding targetedgenes such as v-rel avian reticuloendotheliosis viral oncogene homolog A, tumor protein 53, mitogen-activated protein kinase 1, and C-X-C motif chemokine ligand 8.^[20]^ The gene ontology and kyoto encyclopedia of genes and genomes indicated that Huashi Xuanfei decoction may be mainly acting on several pathways including the IL-17 signaling pathway, tumor necrosis factor signaling pathway, NF-κB signaling pathway, and so on.^[20]^

The epidemic toxins blocking the lung pattern is generally considered more serious than cold-dampness stagnating in the lung pattern with the mail clinical manifestations as fever with red face, cough with little yellow and sticky sputum or blood-stained sputum, nausea and loss of appetite, and lassitude. According to the symptoms, Jiedu Xiefei decoction was formulated with the main mechanisms of detoxifying dampness, cooling blood, and dispersing blood stasis. The primary basis of the Jiedu Xiefei decoction was Qingying Decoction in “Item Differentiation of Warm Febrile Diseases.” Raw Rhubarb (Sheng Da Huang) was a major ingredient in Jiedu Xiefei decoction. It has several positive therapeutic effects such as purging and attacking accumulation, clearing heat and purging fire, cooling blood, and detoxification.^[21]^ The in vivo experimental result showed that raw Rhubarb has positive therapeutic effects on pneumonia, significantly reducing the concentration of pro-inflammatory factors and improving lung disease.^[21]^

As the treatment progressed, all the patients transformed into the lung and spleen qi deficiency pattern. During the convalescent stage, the third prescription named Jianpi Bufei decoction was administrated with the main functions of balancing immune response, nourishing qi, strengthening spleen and lung, and removing residual toxins. The primary basis of the Jianpi Bufei decoction was Shengmai powder in “Origin of Medicine” and Sijunzi decoction in “Prescriptions People’s Welfare Pharmacy.” The Raw Radix Astragali (Sheng Huang Qi), Radix Pseudostellariae (Tai Zi Shen), and Radix Adenophorae (Nan Sha Shen) in Jianpi Bufei decoction all have a long history of medicinal use for providing significant protection against heart, brain, kidney, intestine, liver and lung injury.^[22]^ Modern pharmacological research has reported Radix Adenophorae may enhance the immune function and protect against exogenous pathogens by activating macrophages so as to achieve antivirus action.^[23]^

To obtain an overview of the symptoms profile in 7 periods, the PLS-DA algorithm was applied to data analysis. The PLS-DA algorithm is one of the most commonly used linear algorithms for developing classification models.^[24]^ It focuses on the differences among samples from different classes and operates by splitting the hyperspace of the variables.^[24]^ Although many nonlinear algorithms have been developed to capture complicated relationships, it has been demonstrated that the marginal gain from complicated models is typically small compared to the predictive power of simple models.^[25]^ Moreover, the results of linear models are easier to explain than nonlinear models. The result showed that the 2 groups can be significantly distinguished through the use of the PLS-DA algorithm.

This study has some limitations. First, on account of the prospective property of this study, there were limited patients with confirmed COVID-19. The follow-up time was short and only the confirmed cases in Zhejiang province of China were included. It would be better to include more patients in other provinces, and even in other countries to get a more comprehensive understanding of the effect of TCM prescription through longer-term and larger-scale clinical practice. Second, the patients in the TCM group were screened based on pattern differentiation, but the patients in the control group were not. As a subsequence, some TCM symptoms in 2 groups were not unbiased at baseline. But even more important, the baseline information including gender, age, heart rate, and respiratory rate were all unbiased at baseline between the 2 groups. Third, with respect to the compliance of patients in 7 periods, only 24 noninvasive clinical symptoms were monitored. On the basis of this study, further studies will be carried out to clarify the differences in laboratory results such as leucocytes, neutrophils, lymphocytes, alanine aminotransferase, and aspartate aminotransferase. Besides, the potential molecular mechanisms of TCM prescription should be further investigated.

## 5. Conclusions

This study provided straightforward and comprehensive evidence of the value of TCM prescription for treating patients with COVID-19. The advantage of TCM prescription in promoting recovery of the clinical symptoms, especially fever, poor appetite, and expectoration, was demonstrated. It is expected that this study can provide a more detailed understanding of the efficacy of TCM prescription in dealing with COVID-19.

## Acknowledgments

The authors are grateful to the traditional Chinese medicine (TCM) physicians in First Affiliated Hospital of Wenzhou Medical University, First People’s Hospital of Wenling, Traditional Chinese Medicine Hospital of Lishui City, People’s Hospital of Quzhou City, People’s Hospital of Ruian City, Central Hospital of Yiwu City and Fourth Affiliated Hospital of Zhejiang University School of Medicine for their pattern differentiation, severity assessment, treatment, and TCM symptoms scoring. The patients who participated in this study is gratefully acknowledged.

## Author contributions

**Methodology:** Feihua Huang, Ying Zhou, Cheng Jiang, Yifan Wang.

**Writing – original draft:** Feihua Huang.

**Data curation:** Jinguo Cheng, Xiaoyang Lin, Liping Chen, Chunxian Peng, Guangqiang Pan, Xiaodi Chen, Li Yin.

**Conceptualization:** Kequn Chai.

**Writing – review & editing:** Kequn Chai.
